# Color-Blindness

**Published:** 1887-09

**Authors:** 


### COLOR-BLINDNESS.

A bill is now pending before the Legislature of Georgia requiring the railroads of this State to have all their employés connected with the running of trains examined for that defect of vision known as color-blindness.

It provides that the respective railroad companies shall be held responsible for the payment of a fee of $5.00 for each examination by a medical examiner to be appointed by the Governor of the State.

The great importance attached to the proper understanding of the signals displayed with various colors has led to action in this matter in different sections of the United States by provisions of various kinds, being in some instances under State authority and in other cases by railroad corporations.

The interests of the traveling public and of the railroad companies run parallel in any measures looking to the protection of passengers from injury by accidents to the trains from preventible causes; so that no steps should be neglected which may conduce to the greater security of travelers on the cars of their lines.

In some respects, the advantage of stockholders in railroad companies, as regards their dividends, may prove detrimental to the people who are subjected to their exactions, but in the matter of effecting the safest transportation of passengers over railroad lines, the corporation must look to the welfare of the traveler for its own interest, as the failure to secure efficient officials in the train service must, in the event of the loss of life or bodily injury to any passenger from such mishaps, lead to an action for damages for the injury received. It behooves, therefore, the railroad companies to provide against the casualties likely to ensue from color-blindness in their employés; and when the facts are brought to the attention of those in authority on the different lines in Georgia, we doubt not that all due circumspection will be exercised in selecting their men for the responsible positions, requiring a recognition of colors in signal lights, boards or flags along the tracks.

Legislation upon matters which come strictly within the domain of a corporation would not seem to be requisite, unless there should appear good and sufficient evidence of indisposition or incapacity to accomplish the end in view.

In this case the railroad corporations may be relied upon to protect the traveling public, and ought to be left to do it in their own way.

### ITEMS.

DR. H. V. M. MILLER, of this city, is spending the summer in Asheville, N. C.

DR. JOHN F. LANCASTER, of Walden, Ga., was married a few weeks since to Miss Tinsley, of Macon.

ONE of Atlanta's greatest needs is a first-class city hospital.

DR. T. S. HOPKINS, of Thomasville, is spending some time in Murphy, N. C. He will go from there to Washington to attend the International Medical Congress.

DR. A. W. CALHOUN, of this city, will attend the International Medical Congress, and will go from there to New York, where he will remain until October 1st.

THE amount of money the City Council throws away every year on private institutions would go a long way towards supporting a first-class city hospital.

THERE is a bill now pending in the State Legislature of Georgia making it a misdemeanor for physicians or surgeons to disclose professional secrets. It has been favorably reported by the committee, and it will doubtless become a law, as it ought to.

DR. N. A. RANDOLPH, of Philadelphia, a professor in the medical department of the University of Pennsylvania, and one of the editors of the *Medical and Surgical Reporter*, was drowned on Sunday, August 21st, at Atlantic City, N. J., while surf-bathing, by being carried out by the under-tow.

AUGUSTA, with less than one-half the population of Atlanta, has two well-organized public hospitals. Atlanta is without any public hospital, except the Benevolent Home, and the capacity of that is about thirty patients. There is room here for some public-spirited citizen to immortalize himself and do the sick-poor great good by taking this matter in hand and establishing a first-class public hospital that will be a pride to the city. Who will do it?

AUGUSTA pays her city physicians $100 per month and furnishes them medicines for the sick-poor *free*. Atlanta pays her city physicians $25.00 to $36.00 per month and requires them to furnish all medicines needed. Augusta pays to city physicians a total of $3,600 a year. Atlanta pays for the same a total of $2,440.

THE October number of THE JOURNAL will contain a valuable article from the pen of Dr. Virgil O. Hardon, of this city, entitled "Some Observations Upon Pelvic Cellulitis." This article will embody some new views upon the pathology and treatment of this class of diseases which will render it worthy of careful perusal by all our readers.

THE STATE ANATOMICAL BOARD.—This board, created by the Candler bill, was organized in this city, on the 23d instant, by the election of Dr. Thomas R. Wright, of Augusta, President; Dr. F. W. McRae, of Atlanta, Secretary, and Dr. W. S. Elkin, of Atlanta, Treasurer. Atlanta was made the chief distributing office of the board. It is thought that a sufficient amount of dissecting material will be furnished the colleges by this bill, and that there will no longer be any excuse for grave-robbing in Georgia. The officers are all good men and may be relied upon to carry out faithfully the provisions of the law.

WARN YOUR PATIENTS BEFORE RAISING YOUR FEES.—A French law journal reports an unsuccessful suit of a physician to recover the amount of his bill for a confinement. The bill was for 200 francs, and the husband acknowledged the services rendered, but refused to pay more than 100 francs, which was the amount that he had paid the physician for attendance on his wife in previous confinements. The court decided that he was indebted to the extent of only 100 francs, as the physician had not notified the patient of the increase in his charges.—*N. Y. Medical Record.*

THE BERGEON TREATMENT ABANDONED IN FRANCE.—The treatment of the affections of the respiratory organs by rectal injections of gases is only an advertising pamphlet, endeavoring to load the druggist and doctor with a lot of apparatus that will be just so much dead stock in a short time. There is a popular craze on this subject, which is being utilized by manufacturers and some members of the medical profession, who will reap a rich harvest while the craze lasts. The results which are claimed for this treatment can be obtained by other means and remedies, which have been proved. Late news from Paris says the Bergeon treatment has been abandoned because the results were unsatisfactory.—*Pacific Record*, *August 15th*, *1887*.

LAPAROTOMY FOR PUS IN ABDOMINAL CAVITY.—Dr. Virgil O. Hardon, of this city, performed a successful laparotomy on the 31st of July. The case was one of pelvic abscess, which extended upward into the abdominal cavity nearly to the umbilicus and formed a tumor as large as a fœtal head. The abdomen was opened by an incision two inches in length, and through this the pus-sac was incised, and about a quart of fetid pus evacuated. The cavity was then washed out with carbolic acid and a drainage-tube inserted. The abdominal cavity was also washed out with a carbolic solution and a second drainage-tube inserted into it. The edges of the pus-sac were then stitched to the edges of the abdominal wound. The abdominal drainage-tube was removed on the second day. The pus-sac was washed out daily with carbolic acid. The temperature, which for several days before the operation had stood at 101, rose at no time higher than 101.6, and on the third day became normal and has remained so ever since. The patient is now convalescent, and the pus-sac is rapidly filling up by granulation.

THE TREATMENT OF PAIN BY ANTIPYRIN.—Professor Germain Sée has made a further communication to the Academy of Sciences on the treatment of pain by antipyrin (*The Lancet*). He now advises that the drug be administered by hypodermic injection in doses of eight grains in the same quantity of water. The injection is followed by a disagreeable feeling of tension which lasts a few seconds, and when this passes off the pain whatever may be its cause, at once remits. It has the great advantage over morphine of giving rise neither to vertigo nor vomiting; nor does it produce either the somnolence or the artificial stimulus which lead to morphinomania. Lastly—and this is the great point—to its sedative effect it adds a curative action which morphia does not possess. The diseases for which Professor Sée would more especially recommend the substitution of antipyrin for morphine are numerous—acute rheumatism, acute gout, herpes zoster, lumbago, ataxia; also in hepatic and nephritic colic, angina pectoris, and acute asthma. " But there is no morbid condition in which it does not advantageously replace morphia, and as we gather experience we shall see antipyrin used in its stead, and so become the preservative against chronic morphia poisoning."—*N. Y. Medical Record, August 20, 1887.*

THE ADMINISTRATION OF CARBONATE OF LIME AS A MEANS OF ARRESTING THE GROWTH OF CANCEROUS TUMORS.—Nearly twenty years ago Dr. Peter Hood published a communication on the value of carbonate of lime, in the form of calcined oyster-shells, as a means of arresting the growth of cancerous tumors. In *The Lancet* (May 7, 1887) he publishes a second communication on the same subject, in which he states that although his opportunities for employing it in suitable cases have not been large, the results which he has attained through its use have been very satisfactory. He refers to several cases in which a persevering use of the calcined shell-powder arrested the growth and pain in tumors undoubtedly of a cancerous character. Dr. Hood urges the persistent and fair trial of this remedy in cases of cancer, where the nature of the affection is early recognized. It can do no possible harm, it need not interfere with other remedies for the relief of pain, its action can be referred to an intelligible and probable hypothesis, and it has been of utility in a sufficient number of cases for warranting us in reposing some confidence in its use. An advantage of the treatment is that the remedy may be readily prepared at home by baking oyster-shells in an oven, and then scraping off the calcined white lining of the concave shell. The substance thus obtained is to be reduced to a powder, and as much as will lie on a silver quarter taken once or twice a day in a little warm water or tea.—*N. Y. Medical Record.*

SWALLOWING GLASS.—Dr. J. C. McMillan, of Marion, S. C., reports, in the form of a reprint, the case of a child ten months old, who had been playing on the floor where a lamp-chimney had been broken, and had swallowed some of the fragments The mother had been in the habit of giving it pieces of ice frequently, and doubtless the infant took to eating the glass for the ice. The child was seen by the writer the day after the glass had been taken into the stomach. There seemed to be very little physical disturbance, and aside from a slight rise of temperature, 99½°, the little patient seemed to be enjoying its usual health, although it had been for two days constipated. The treatment consisted in rest, castor-oil, flaxseed and slippery elm. A few hours after the first dose of oil, the glass began to pass. A dose of oil was given every day for four days, the glass continuing to pass each day. The mother did not collect the glass on the first day, but on the three days following seventy pieces were collected, varying in size from one-fourth to three-fourths of an inch in length. After this the patient seemed to get along very nicely. Notwithstanding that part of the glass remained in the intestinal canal for six days, no symptoms of inflammation, ulceration or obstruction occurred.—*N. Y. Medical Record.*

DEATH BY DECAPITATION.—It is probably due to the fact that the prescribed methods in France for executing the death-penalty offer peculiar facilities to scientific investigation, rather than to any predilection for the grimmest of all studies in experimental physiology, that our French confréres have reported so many observations on the nervous and other phenomena of the first few seconds after sudden death. The latest observations by Drs. Regnard and Loye, in the *Progrés Médical* of July 9, and the *British Medical Journal* of July 23, enforcé the fact that death by the guillotine is quite another thing than that method employed judicially by the Anglo-Saxon race. The examination was made of the head and body of a convict immediately after his decapitation by the guillotine. The prisoner was calm to the last, and not pale, even when his neck was fixed ready to receive the fatal knife. Two seconds after decapitation the cheeks were still rosy, the eyes wide open, with moderately dilated pupils, the mouth firmly closed. No fibrillary contractions could be observed. When a finger was placed close to one eye, no change of expression took place; but on touching an eye or the tips of the lashes, during the first five seconds, the lids closed just as in life. This reflex action could not be elicited from the sixth second after decapitation. The jaws were tightly clenched, and could not be opened by manual force; no similar muscular contraction could be detected in the trunk or extremities. One minute after death the face began to turn pale, the trunk remained flaccid, the carotids continuing to throw out blood remaining in the circulatory area. At the end of four minutes the face was quite pale, the upper lids were half closed, the jaws less firmly clenched than before. Irritation of the cut surfaces of the spinal cord failed to produce reflex movements either in the trunk or on the face. For twenty minutes there was no change; then the necropsy was begun. There were signs of old pleurisy and alcoholism. The heart beat actively. On opening the pericardium, the ventricles and auricles continued to pulsate for twenty-five minutes; the former then ceased to beat, but the auricles went on for forty minutes longer.

4

Thus the heart beat for an hour after decapitation. Then its chambers were laid open; the left ventricle was firmly contracted, the right relaxed. There was emphysema at the edges of the left lung, as is nearly always observed after death by the guillotine. There were bubbles of air in the vessels of the *pia mater*, and much air in the subarachnoid space. The knife had passed through the lower part of the fourth cervical vertebra. These researches show that not a trace of consciousness remains two seconds after beheading; that reflex movements of the cornea can be excited for a few seconds; that the heart may beat for an hour, the auricles continuing to pulsate alone for over half that period, and that, putting aside the reflex movements of the eyelid, the contraction of the jaws and the jets of blood from the carotids, it seemed in this case as though a corpse had been decapitated, so inert were the remains of the convict. The entry of air into the inextensible and incompressible cranial cavity, after the escape of blood from its vessels, was only to be expected. Drs. Regnard and Loye note how calm and free even from physiological death-struggle symptoms is death by the guillotine. There is not even asphyxia; death is rather due to inhibition similar to that described by M. Brown-Séquard in animals who succumb to certain irritations of the nervous system.—*Boston Medical and Surgical Journal.*

HYSTERO-EPILEPSY IN THE MALE.—Professor Mendel showed a patient at the meeting of the Medical Society of the 13th ult., the subject of so-called hystero-epilepsy. The man was a master handicraftsman, æt. 45, who every day, from 9 o'clock in the morning till 6 the following morning, was deaf and dumb, and between six and nine spoke and heard in a perfectly normal manner. There was no hereditary tendency to nervous disease. In consequence of an injury some years previously, pains came in the right arm which proceeded to contractions. The contractions disappeared, however, of themselves in the course of a few years to re-appear in the left arm. Since 1872 the patient had suffered, in consequence of a psychical disturbance, from epileptic attacks. The present illness dated from March, 1886, when, at the termination of one of his epileptic attacks, he suddenly lost the sense of hearing and the faculty of speech. This condition lasted fourteen days, after which he could hear and speak at intervals. At first the deafness and dumbness came on at eight in the evening and lasted till six in the morning; then they came on at 5 p. m., then three, and gradually earlier in the day until at last they came on at 9 a. m., but they always ceased at 6 a. m. This was the condition from June, 1886; from six to nine the patient had complete use of the faculties of speech and hearing; from nine he could neither speak nor comprehend speech until six the following morning. The mental condition was quite normal; the patient superintended his business, went journeys, reckoned accounts, and by means of writing conversed with those about him. Examination of the ears and larynx showed a perforation of the right tympanum, and some trifling changes in the vocal cords, but neither prevented him speaking and hearing normally between 6 and 9 p. m. As soon as the patient was touched in a particular spot in the right forearm near the wrist-joint, he fell into violent convulsions, accompanied by some disturbance of consciousness. When he was again touched on a particular spot in the neck, the convulsions ceased. Professor Mendel described the affection as hystero-epilepsy, and remarked that in the whole literature of the subject only one similar case was recorded. He laid less stress upon the spasm that could be voluntarily produced than upon the regular returning and disappearing hysterical deaf-mutism. He hoped to cure the patient quickly by hypnotism *à la suggestion*, a method of treatment as mystical as the disease for which it was to be applied.—*Berlin Correspondent Medical Press, August 10, 1887.*

THE DANGERS OF ANTIFEBRIN AND ANTIPYRIN.—Two articles have recently appeared in the Hungarian medical journal, *Gyógyázat*, in which the profession is warned that acetanilide, or antifebrin, as it is called, though it is not without value, is an uncertain and indeed a dangerous antipyretic. Dr. Géza Dulàckska finds, after giving the drug a prolonged trial in the hospital, that small doses may bring down fever, but that in some cases even three-grain doses produce violent sweats, prostration, and hemorrhage or cyanosis. It does not appear to him to exert any favorable action on the course of the disease itself, being thus inferior to salicin, resorcin and ichthyol. If given at all in fever, its action requires to be very carefully watched. In certain neuroses, however, Dr. Duláckska has found it very useful, and as the doses required were smaller, he is disposed to recommend its employment in such cases, as it appears to be free from danger. In a case of severe trigeminal neuralgia, in which quinine, gelsemium and morphia had produced but little improvement, three grains of acetanilide were given night and morning without effect; seven grains and a half were then ordered, divided into ten powders, one to be taken every two hours. This produced a great improvement, and after ten days of this treatment the cure was complete. No unpleasant effects beyond some nocturnal perspiration showed themselves. Two other cases, where severe pain was associated with meningo-myelitis and syphilitic tabes respectively, which had been treated in various ways unsuccessfully, were greatly benefited by acetanilide, no unpleasant effects being produced. Dr. Biro gave acetanilide in eighteen cases of various kinds, including typhoid, croupous pneumonia, pleurisy, polyarthritis, tuberculosis and phthisis, in doses varying from a grain and a half to forty-five grains per diem, and was much struck with the uncertainty of its action, an effect being sometimes produced by a grain and a half, while at others ten or eleven grains had no effect at all. Most of the patients complained of chilliness, while cyanosis and irregularity of pulse occurred in several cases. In one case, where seven grains and a half had been taken, there was a severe rigor, followed by cyanosis, sweating and an irregular, thready pulse, the symptoms being so alarming that recourse was had by brandy and hypodermatic injections of ether. In another case, in which acetanilide had been given for eleven days, similar symptoms presented themselves, together with clonic spasm of the lower extremities, reminding one of the symptoms in anilin poisoning. Altogether, Dr. Biro thinks that the evil effects of acetanilide are so great that it cannot be recommended for general adoption.

Two Scandinavian physicians have recently written on the use of antipyrin, the apyretic properties of which they unreservedly admit to be the rule. Some exceptions, however, were observed. Thus Dr. Wetterdal, of Upsala, found in one case a rise of temperature during the first hour after a thirty-grain dose of more than 1° F. Rigor and collapse never occurred in any of his cases. Copious sweating, however, was observed at times, over which atropine exerted no control. A rash with bluish-red spots on the face, as described by Wising, was also occasionally noticed. In some cases cinchona was advantageously combined with antipyrin. Dr. Laache, who made a special study of the action of antipyrin in phthisis, found that, though as a rule it brought down the hectic very satisfactoilry, in one instance it produced at first at least a contrary effect, the temperature rising very markedly after a thirty-grain dose. It afterward fell gradually, reaching a point decidedly below the normal. Simultaneously with the rise a rash appeared, and the patient complained of a burning sensation in the mouth and eyes and along the œsophagus. Curiously enough, the patient had previously taken antipyrin for a longer period without having experienced any unpleasant effects. The sample could not well have contained any poisonous admixture, as other patients took it with impunity.—*Lancet Medical News, August 20th, 1887.*

REMOVAL OF TWENTY-SIX CALCULI BY LITHOTOMY.—At the meeting of the Imperial and Royal Society of Practitioners in Vienna, Dr. Dittel read notes of a remarkable case of lithotomy which he had performed. The patient was a great wine-drinker. Fourteen years before operation he suffered from pains in the lumbar region and hæmaturia. The pains lessened gradually, but again became severe. After suffering great agony for six weeks he consulted Dr. Dittel. The presence of numerous calculi in the bladder was easily discovered by the use of the sound. The patient looked sickly, but was rather fat. The urine was putrid and gave evidence of diphtheritic inflammation of the bladder. Washing out with solutions of carbolic acid and chlorides of zinc hardly improved the condition of the bladder, though its capacity was increased. The case did not appear suitable for any form of lithotrity. Dr. Dittel considered that the suprapubic operation was unadvisable, owing to the corpulence of the subject and the difficulty of keeping the wound aseptic. He determined, therefore, upon performing perineal lithotomy. The operation was performed on May 29th. During the whole of its course a solution of salicylic acid was made to run into the bladder through the urethra. Twenty-six calculi, "which could not exactly be called small," were removed. The patient was progressing favorably on June 10th, and the temperature had been high only on one occasion.—*British Medical Journal, July 9, 1887.*

A NEW TEST FOR MORPHINE.—A novel and very beautiful test for the presence of small quantities of morphine ($\frac{1}{200}$ gr.) has recently been suggested. To the solution to be tested add a few drops of strong sulphuric acid and about the same quantity of a solution of sulphate of sodium. Heat the mixture in a porcelain capsule, and directly it begins to give off sulphuric vapor; cool it suddenly, when it assumes (if morphine be present) an intense violet coloration. If the mixture be further heated, it turns brown, and when cooled, the addition of a few drops of water determines a vivid red coloration, which turns a pale green if more water be added. If at this stage an equal bulk of chloroform be poured into the mixture and well shaken, the chloroform becomes of a bright blue color.—*British Medical Journal, July 9, 1887.*

INFANTILE DYSPEPSIA.—At a recent meeting of the *Academie de Medecine* M. Hayem read a paper on the treatment of dyspepsia in infancy, and especially that form of it which is accompanied by green-colored diarrhœa. He points out that the green color seen in diarrhœa of infants at the breast is due to a substance produced by a particular bacillus. He maintains that the disease is contagious, and that the germs deposited on the napkins from the stools are contaminating agents. All linen or flannel, therefore, which is soiled either by vomited matter or dejecta should be removed as quickly as possible, and plunged into pails containing a one per cent. solution of corrosive sublimate. A teaspoonful of a two per cent. solution of lactic acid should be given to the infant a quarter of an hour before putting it to the breast. From five to eight doses are given in twenty-four hours which represents about 40 to 60 centigrammes of pure lactic acid.—*British Medical and Surgical Journal, July 9, 1887.*

AN EMULSIVE FOR CASTOR OIL.—A great many vehicles have been proposed for the purpose of mitigating the disagreeable insipidity of this useful but nauseous medicine. A French contemporary has recently suggested casein as a good emulsive. It must be chemically pure, but a little bicarbonate of sodium and powdered sugar should be added. Half an ounce of castor oil is triturated or shaken up with a drachm and a half of laurel water, and sufficient casein to form a staple emulsion, distilled water being added to make up three fluid ounces.—*Medical Press.*

Treatment of Piles by Dilatation.—In the *Gaz. des Hopitaux*, M. Verneuil publishes a note on the treatment of piles by dilatation. According to the author, ninety-eight cases out of a hundred may be radically cured by this simple process. The duration of the treatment scarcely ever exceeds eight days, during four of which the patient remains in bed, and during the remaining four days in his room. Piles of six, eight, ten, twelve and fourteen years' existence have been completely cured in this manner. Even in cases in which the disease is complicated with true rectal prolapsus, dilatation should be had recourse to. During fifteen years that the author has practiced this method, he has not met with one unsuccessful result. He prefers the speculum to the digital method of dilatation.—*Medical and Surgical Reporter.*

The Remedial Value of Blood-letting.—It has often seemed a matter of regret that a remedy of such unquestionable power as blood-letting should, from former abuse, be reckoned by many as among the things of the past, and that it should have run the risk of being denied all virtue because of some inherent faults, which, however, are quite capable of compensation. Its very power, and the exact results which in fitting cases attend its employment, doubtless led to its indiscriminate use, and, inasmuch as it is spoliative in its nature—a power fraught, it may be, with the greatest evil—it is not difficult to see how readily it might be abused.

Dr. J. A. Macdougall, of Carlisle, in the July number of *The American Journal of the Medical Sciences*, makes an able plea for its more frequent use, and cites clinical evidences of its value. He points out that with that greater skill which is undoubtedly ours, with that more intimate acquaintance with physiological and pathological processes, we are better able to judge the exact capability of such a remedy, and when we recognize in it the power to modify the distribution of the blood, and to diminish pressure within the vascular system, then we are moving on such lines as are well fitted to guide us in its employment. That it can do more than these things is probable. That it does act as a derivative, that it is a powerful though dangerous sedative, and that its employment facilitates the action of other remedies, is all possible, and although he believes that few would incline to employ it for such ends solely, its possession of such potentialities may render it of wider service than we anticipate when we use it.

ASPIRATION OF THE STOMACH IN DRUNKENNESS.—In the *Dublin Journal of Medical Science*, Mr. George Foy relates the following case: One night in November, 1884, he was called to a man reported to be dying. He had taken a large quantity of whisky and claret, and for two hours his drunken companions had been trying to rouse him, but had totally failed. When seen he was quite insensible; extremities cold, face livid, pupils widely dilated, respiration and pulse almost imperceptible. Treatment was commenced by opening the median-basillic vein of the left arm, when, by rubbing the forearm briskly, the blood gradually flowed until sixteen ounces were drawn off. In the meantime the fine trocar of an aspirator was pushed through the abdominal wall in an upward, backward and outward direction, just at the sternal end of the eighth rib. The cannula was attached by the ordinary method to an exhaust bottle, and on turning the cock a stream of claret-colored fluid flowed into the bottle.

Very soon the man was able to speak, and soon recovered. The author saw no good in giving apomorphia in this case, as it has no emetic action when sensibility is quite deadened, while the respiration was too feeble to allow a stomach tube to be introduced; so that there seemed no chance for the patient unless aspiration was attempted.—*New York Medical Record*, *July* 23, 1887.

A NOVEL DEPARTURE IN ADVERTISING.—Believing that the advertising of medicinal preparations often fails of its purposses, viz., to clearly and intelligently present to physicians their special advantages, pharmacal or therapeutic, on account of the fragmentary and imperfect manner in which the facts are usually conveyed in such advertisements, Parke, Davis & Co. propose to inaugurate rather a novel departure in advertising. It is their intention to publish in the advertising pages they occupy in medical journals a series of what they term plain talks to physicians, in each issue taking up a certain class of preparations and pointing out the reasons why they deserve to be prescribed until all their preparations shall have thus been presented. The excellence of the products of this house is well known, and it is to be presumed that their long experience in the manufacture of medicines will enable them to say in these informal talks something of real interest and benefit to their medical friends.

THE INJURIOUS EFFECTS OF BLISTERS.—Dr. Wyss, of Geneva, is a strong opponent of the employment of blisters, which he considers an unnecessary cruelty to the patient, and an injury, in that they prevent sleep, disturb the function of urinary secretion, and often give occasion to obstinate cutaneous eruptions. This same stand was taken by Dr. Goldmann, now of Galveston, in a letter to *The Medical Record* from Monterey, Mexico, in January,

1884. In this letter the author gave his reasons for his objections to blistering, and stated his belief that all the good effects of vesication could be obtained by other means which do not possess its drawbacks.—*N. Y. Medical Record, Aug. 27, 1887.*

DIPHTHERIA IN ANIMALS.—The report of Dr. George Turner, which has just been issued by the Local Government Board, on the subject of the existence of a disease in animals bearing a striking analogy to diphtheria in human beings, is a step further on the path which was first started when the possibility of infection being conveyed from animal to man and *vice versa* was recognized. From Dr. Turner's observations it would seem that a certain reciprocity exists between man and animals in the intercommunication of this particular disease. Epidemics of diphtheria in human beings have been accompanied by special forms of disease in poultry and domestic animals, and in certain cases the latter has preceded the former. In this way Dr. Turner is disposed to explain outbreaks of diphtheria under circumstances which would seem to render infection from human beings impossible. Now that the results of Dr. Turner's painstaking observation have been made public, further evidence bearing on the subject will doubtless be forthcoming, and light will be thrown on a matter which, however probable, is so far based only on a concatenation of probabilities. Patient and conscientious workers in this field of observation have a rich harvest before them, and the progress already accomplished cannot fail to act as a powerful incentive to further research.—*Medical Press, Aug. 10, 1887.*

SAGACITY OF A CHEMIST.—A case has recently been reported in Paris in which a chemist acted with consummate judgment, so much so that it may be hoped that the course of action he adopted upon the occasion will be followed as a precedent by his *confréres* in the trade, and be borne in mind and imitated when circumstances seem to require it. A flower girl, smarting under the infliction of being deserted by her lover, who had avowed his intention to marry another woman, made a point of attending the marriage ostensibly for the purpose of disposing of her floral wares. In the midst, however, of the rejoicing after the ceremony, she suddenly confronted the newly married pair, and quickly withdrawing a bottle from her basket violently threw its contents into the face of each. A terrible scene ensued, and the worst results were anticipated by the assembled guests, the belief being that the young couple would be blinded for life. Instead, however, of this woeful termination to an auspicious gathering, nothing more dreadful happened than what a pocket handkerchief was amply sufficient to relieve. The fluid, owing to the sagacity of the chemist, was only tinted water. The flower girl, in asking for sulphuric acid, betrayed a demeanor which suggested to the shopman the probable use for which the powerful agent was required, and hence a direful tragedy was frustrated; the faces of the two persons still remain unseared, and a woman's revenge for once has been unsatisfied.—*Medical Press, Aug. 17, 1887.*

DEATH OF ONE OF M. PASTEUR'S PATIENTS.—An inquest was held at St. George's Hospital, on August 10th, on the body of Martin Cahil, aged 29, who was severely bitten on the left wrist at Templemore on June 14th by a mad dog. It was stated by Lieutenant Walter Rushmarsh, in whose employment the deceased was, that the dog having escaped, he followed it, and on returning with the dog was himself bitten by it on the right hand, and it subsequently came to his knowledge that the same dog had bitten two of the hounds in a pack belonging to the 18th Hussars, stationed in Cork. Both the dogs died mad. On Saturday, June 25th, the witness, who had previously had his wounds cauterized, started for Paris to undergo treatment at the hands of M. Pasteur. The witness was treated for ten days, at the end of which time he was pronounced cured. The deceased, whose wounds were much worse, was treated for seven days longer, and was told when discharged that his case was still dangerous. Some days after, it was found that he was still suffering from the effect of the bite, and was sent to the hospital, where he died on August 7th. Mr. Russell Coombe, house-surgeon at the hospital, said he treated deceased as an out-patient for five days, at the end of which time he brought a note from his mistress, stating that he could not sleep. At the time the deceased was impressed with the idea that he was going to die, and that he could not swallow any food. Between four and five in the morning of the 6th inst. witness was sent for, and found the deceased fighting with the porter. He was exceedingly violent, and made a noise as if imitating some kind of an animal. He died at ten o'clock the next day. Witness was unable to say what was the cause of death. The jury found a verdict that the deceased died through being bitten by a dog, and that the bite was accidental.—*British Med. Journal*, *August 20th*, *1887*.

THE MUMMY OF SESOSTRIS.—The following particulars of the examination of the remains of Rameses II., made by Dr. Maspero at Boula possess a certain interest: "When the bandages were removed, after the lapse of some three thousand years, the body was exposed to view. The summit of the cranium was denuded. The hair, scanty on the temples, was plentiful over the occiput, and was stained of a yellow color. The forehead was low and narrow, the eyebrows prominent, the eye-lashes white and thick, the eyes were small and near together, the nose long and thin, and flattened from the pressure of the wrappers. The temples were depressed, and the cheek-bones well marked; the ears stood out from the side of the head, and were pierced like those of a woman. The mouth was small, the lips thick and fleshy. It was filled with a dark-colored paste, on removing which some friable teeth were visible, which though worn were white and well preserved. The beard and moustache appeared to have been allowed to grow only during the last illness or after death, the hairs being about an eighth of an inch long. The skin was of an ochre color with black stains. Nevertheless the face gave a very fair impression of what it must have been during life, not too intelligent, even rather vulgar, but indicative of pride, obstinacy and domination. The other parts of the body were not less well preserved than the head, but the desiccation of the soft parts had materially affected their outline. The neck was reduced to the diameter of the vertebral column; the chest was broad and the shoulders high. The arms were crossed over the chest and the hands were delicate and stained. The wound through which the embalmers had removed the viscera was extensive, and was situated on the left side. The genital organs had been removed with a cutting instrument. The thighs and legs were emaciated, the feet long, thin and flat. The corpse was evidently that of a robust and vigorous old man.—*Medical Press, Aug. 17, 1887.*

A CASE OF PERITONITIS FROM INJURY.—A case of considerable medico-legal interest has recently been tried in the County Court of Skipton. A boy aged 13, who was attending the grammar school, was squeezed by one of the masters against the desk at which he was sitting. He believed it was done as a punishment; but another boy, who saw it, thought it was intended as a joke. The master in question denied all recollection of the occurrence. The boy said he was trying to stand up at the moment, so that his umbilical region was compressed against the ledge of the desk. He complained of being hurt at the time, and was obliged to go home and go to bed on account of the pain, and on the succeeding days he was so unwell that, on his return from school, he went to bed each day. A few days afterwards he was persuaded to play at foot-ball, but had to desist in less than a quarter of an hour, as he felt so ill. About the twelfth day after the injury Dr. Wylie was called in, and found that the boy had well-marked local peritonitis in the umbilical region, from which, after a good deal of suffering, he eventually recovered. In the end the jury found that the peritonitis had been due to the squeezing, but that the master had pushed up against him in joke, and not with intent to do him harm, and awarded the damages at £10. An attempt was made to show that the peritonitis must have been caused in some other way, as it was so long in making its appearance; and medical evidence was called to prove that acute peritonitis usually set in within a few hours after the receipt of the injury, but that the time at which it commences must depend upon the nature of the injury. Great violence, no doubt, may produce an exceedingly rapid acute peritonitis, but a lesser degree of violence would produce only a subacute peritonitis, and we should not expect it to make its appearance so soon. After all, there is no evidence to show in this case when the peritonitis did develop; and, judging from the nature of the injury, it seems most probable that its course was a very gradual one, and that it had been in existence for many days before the medical man was consulted.—*British Med. Journal, August 6, 1886.*

THE usefulness of good hypophosphites in pulmonary and strumous affections is generally agreed upon by the profession. We commend to the notice of our readers the advertisement on page 32 of this number. "ROBINSON'S HYPOPHOSPHITES" is an elegant and uniformly active preparation, the presence in it of quinine, strychnine, iron, etc., adding highly to its tonic value.

